# Using Wearable Technology to Predict the Occurrence of Severe Behavior Problems among Neurodiverse Individuals: A Systematic Review

**DOI:** 10.1007/s40614-026-00497-1

**Published:** 2026-03-25

**Authors:** Patrick W. Romani, Sidney K. D’Mello, Robert M. Moulder, Lily N. Berkowitz

**Affiliations:** 1 Department of Pediatrics, University of Colorado School of Medicine, Aurora, CO USA; 2 Institute for Cognitive Science, University of Colorado Boulder, Boulder, CO USA; 3 Department of Psychiatry, University of Colorado School of Medicine, Aurora, CO USA; 4 Present Address: Northwestern University Feinberg School of Medicine, Chicago, IL USA; 5 Ann and Robert H. Lurie Children’s Hospital of Chicago, 225 East Chicago Avenue, Chicago, IL 60611 USA

**Keywords:** Biometrics, Machine learning, Neurodevelopmental disability, Prediction, Severe behavior problems

## Abstract

Severe behavior problems (SBPs) exhibited by individuals with neurodevelopmental disabilities (NDD) can produce challenging and potentially dangerous situations. Although the field of behavior analysis has access to effective behavioral assessment and treatment methodologies, the risks associated with serving individuals with NDD engaging in SBPs remain high. Advances in wearable sensing, artificial intelligence, and machine learning offer potential support for behavior analysts working with individuals engaging in SBPs. Thus, researchers have begun studying physiological and behavioral signals (i.e., biometrics), such as heart rate or bodily motion, and their predictive relationship with SBPs. The current systematic literature review summarizes 13 peer-reviewed articles that studied predictive relations between biometrics and SBPs. We highlight commonalities, differences, and limitations among these studies. In particular, although some studies claim to predict the occurrence of SBPs over 30 s in advance of their occurrence, methodological concerns reduce the veracity of these claims. We propose short-term and long-term research questions to move this line of research forward.

Neurodevelopmental disorders (NDD), comprising diagnoses such as attention-deficit/hyperactivity disorder autism spectrum disorder (ASD), intellectual disability, and learning disability, affect many areas of daily adaptive functioning, such as communication and daily living skills (Antshel & Russo, 2019; Holden & Gitlesen, 2006). Yang et al. (2022) studied the prevalence of NDDs between 2019 and 2022. They found that 8.5% (attention-deficit/hyperactivity disorder), 2.9% (ASD), 1.4% (intellectual disability), and 6.4% (learning disability) of the U.S. population meets criteria for these NDDs. These data have significant implications for policy makers, researchers, and clinicians, alike, because the treatment of individuals with NDDs tends to be intensive and requires a person-centered approach (Marquez-Caraveo et al., 2021).

Neurodiverse individuals tend to seek treatment for mental and behavioral health symptoms, such as irritability, more often than their typically developing peers (Kroll et al., 2025). In fact, diagnoses of NDDs tend to be comorbid with symptoms of irritability, which sometimes lead to the development of aggressive behaviors (Esteves et al., 2021; Eyre et al., 2019). These severe behavior problems (SBP; e.g., aggression, self-injury) often require intensive treatment because of their adverse impact on community, school, and home functioning. Needless to say, there is a major need to identify effective ways to reduce SBPs.

Treatment based on the principles of behavior analysis represents one evidence-based approach for targeting SBPs among individuals with NDDs (Lindgren et al., 2020). These behavioral treatments are based on the results of functional behavior assessment (FBA; Roane et al., 2016), which consists of three broad categories of assessment: indirect, direct, and experimental (Tarbox et al., 2009). Although each category of FBA is helpful, only the experimental analysis manipulates antecedent and consequence variables in a way to identify behavioral function. That is, a determination of whether SBPs are maintained by access to attention, access to preferred items, escape from task demands, or are reinforced independent of the social environment (i.e., automatic reinforcement). Behavioral treatments based on the results of FBA tend to be more effective than those not based on FBA (Herzinger & Campbell, 2007), and this model of behavioral assessment and intervention has been validated through randomized controlled trials and consecutive controlled case series (Ghaemmaghami et al., 2021; Greer et al., 2016; Lindgren et al., 2020).

Over time, research consistently shows these treatments to be highly effective (Greer et al., 2016; Lomas Mevers et al., 2018). But relapses are common (Nevin & Wacker, 2013). Briggs et al. (2018) showed that treatment relapse, or the acute increase in the occurrence of SBPs following changes to treatment conditions, occurred in 19 out of 25 applications of behavioral treatment. Although most forms of treatment relapse are a temporary phenomenon (Fisher et al., 2022), the risk of injury to the client or caregiver when reexposing the SBP to reinforcement contingencies exists. There are few studies showing the conditions under which injury to clients or caregivers, such as behavior technicians or parents, occur when implementing behavioral treatment (Ruby et al., 2023). Although the risk of injury when serving individuals with NDDs engaging in SBPs is high, there is no clear research to support just-in-time intervention—defined as the delivery of functionally relevant supports during brief periods of imminent risk prior to SBP occurrence—to maintain a safe environment for caregivers and their clients when implementing behavioral treatment, particularly during periods of treatment relapse (Goodwin et al., 2019). Consider an example of a client engaging in attention-reinforced problem behavior. A caregiver could prompt communication response or even deliver attention contingent on precursor problem behavior if alerted to the risk of SBPs occurring in the next 5–10 s. Just-in-time intervention has supported treatment outcome for individuals experiencing substance abuse (Koike et al., 2025) as well as other mental health conditions (Von Lutzow et al., 2025). The field of behavior analysis would likely benefit from similar intervention approaches (Gifford & Valdovinos, 2023).

To address this challenge, researchers are increasingly focused on identifying antecedent biomarkers of SBPs that may precede behavioral escalation (Goodwin et al., 2018; Smith et al., 2025; Zheng et al., 2021). One promising direction involves physiological indicators of arousal, which are known to correlate with SBP occurrence (McDonnell et al., 2015). Physiological arousal—manifesting in changes in heart rate, bodily movement, or electrodermal activity—can be continuously monitored using wearable technologies (e.g., smart watches; Taj-Eldin et al., 2018). This opens the door to detecting *precursors* of SBPs that may not be captured through traditional FBA methods, which emphasize observable environmental events.

This evolving line of research provides the foundation for a conceptual framework in which SBPs are seen as the outcome of dynamic biopsychosocial processes (Goodwin et al., 2018; Imbiriba et al., 2023; Zheng et al., 2021). In this framework, biological arousal states may function as a motivating operation that alters the reinforcing value of consequences historically associated with SBPs, thereby increasing the probability of SBP occurrence (Laraway et al., 2003). It is important to note that this conceptualization invites the use of machine learning—a set of methods capable of analyzing complex, multivariate time-series data to detect meaningful patterns in physiological signals. Machine learning computational models are well-suited to identify *nonlinear relationships* and *temporal dependencies* in large physiological datasets—enabling the prediction of SBP occurrence, ostensibly before it is observable to the human eye (D’Mello et al., 2022). For instance, accelerometer data have been shown to discriminate between self-injury and non-self-injury in autistic individuals (Cantin-Garside, Kong, et al., 2020; Cantin-Garside, Srinivasan, et al., 2020), and wearable shirts tracking heart rate and respiration have identified physiological changes that precede SBPs (Barrera et al., 2007). Romanowicz et al. (2023) claimed that machine learning models trained on physiological data from smartwatches could predict SBPs in psychiatrically hospitalized children up to 30 s in advance. Although there are counterexamples (e.g., Cheung et al., 2022; McCabe & Greer, 2023), these findings illustrate how machine learning techniques can be integrated into a predictive model of behavior, potentially transforming the management of SBPs through *just-in-time* alerts to caregivers, thereby enhancing both safety and treatment durability (Freeman et al., 1999, 2003).

In summary, the integration of behavior-analytic assessment and treatment procedures with wearable sensors and ML-based predictive modeling introduces a potential proactive dimension to behavioral intervention. This approach can extend current best practices when it comes to the treatment of SBPs by offering real-time data that may inform decision making during high-risk moments (Goodwin et al., 2019). For example, a caregiver could be alerted about the increased likelihood of SBP occurrence and deliver putative reinforcers to reduce the likelihood of SBP occurrence. This approach could reduce the likelihood of SBPs and create safer environments for clients and caregivers alike. Thus, the application of ML to predict SBPs can represent a conceptual advance in behavior analysis, aligning with long-standing goals of increasing treatment effectiveness, generalization, and maintenance (Kimball et al., 2023; Nevin & Wacker, 2013; Nevin et al., 1983). However, the crux of the matter depends on whether the machine learning models can in fact reliably detect SBPs in advance, a claim that has yet to be critically evaluated. Thus, the purpose of the current systematic review is to summarize and critically review existing research on the use of wearable technology to detect physiological signals predictive of SBPs among individuals with NDDs. To our knowledge, this is the first review to examine the intersection of wearable technology, physiological arousal, and machine learning for SBP prediction in this population.

## Method

### Search Strategy and Eligibility Criteria

We followed the Preferred Reporting Items for Systematic Reviews and Meta-Analyses (PRISMA) guidelines when conducting this review (Page et al., 2021). Our literature search took place between May 2024 and March 2025. We searched the PubMed and Ovid databases (i.e., MEDLINE, CINAHL, PsychINFO, Embase) using combinations of the following search terms: autism spectrum disorder, neurodevelopmental disorder, attention deficit hyperactivity disorder AND biosensors, wearable sensors, predicting problem behavior, heart rate, accelerometer. Boolean search strings combined population terms (e.g., autism spectrum disorder, neurodevelopmental disorders) with wearable sensor and physiological measurement terms (e.g., biosensors, accelerometry, heart rate), and behavioral outcome terms related to problem behavior. Truncation (*) and Boolean operators (AND/OR) were used to maximize sensitivity across databases.

**Fig. 1:**
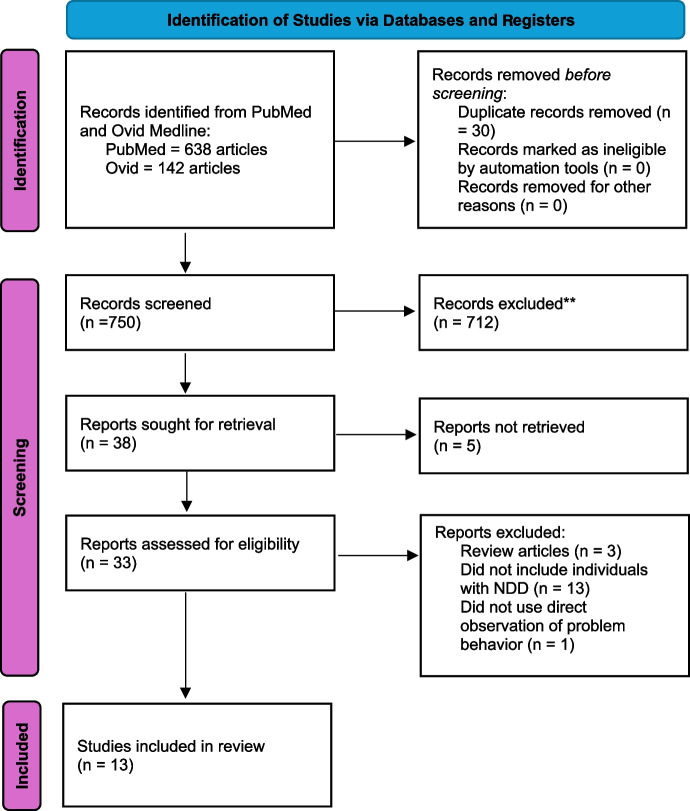
This is mandatory. Please provide

To be included in this review, studies needed to incorporate wearable technology (e.g., heart rate sensors) to make predictions about SBP occurrence, include individuals diagnosed with NDD as participants, and be peer reviewed in English-language journals (see Fig. 1 for a visual depiction of the systematic review process). The initial search returned 780 articles, of which 30 were duplicates and thus removed from the evaluation. The first and fourth authors conducted a title, abstract, and full-text review of the remaining 750 articles. We ensured the reliability of the screening process by both reviewing 33% of the articles (248/750). We agreed to keep or remove 97% of the 248 articles. We retained 36 articles after removing 718 following the title and abstract review. After a full-text review of the remaining 36 articles, we retained 13 and removed 23. Of the 23 articles removed, 3 were review articles, 13 did not include individuals diagnosed with NDDs, and one did not use a direct observation of problem behavior. After identifying these 13 articles, we conducted an ancestral search of each article by reviewing the reference lists. We identified four additional articles that were not retained following a full-text review.

### Data Extraction and Analysis

Each of the 13 articles were coded for: (1) publication outlet and year; (2) participant demographics and setting; (3) specific wearable devices used; (4) data collected during the study; (5) body location of the wearable device; (6) observation arrangement; (7) statistical approaches used; and (8) outcomes. The first and fourth author coded each article and reached 100% agreement. Please see Table 1 for a summary of these data.

**Table 1:** Summary of 13 Studies Included in this Systematic Review

Study	Participants/Setting	Wearable Devices and Cost	Data Collected	Device Location	Observation arrangement	Statistical approaches	Outcomes
Barrera et al. (2007)	3 participants (27–42 years) / Outpatient clinic	VivoMetrics LifeShirt	Freq. SIB; ECG–BPM, respiration, movement, posture, and temp	Chest, abdomen, shoulder blade	Functional analysis of problem behavior	Percentage difference in mean HR	Consistent increase in HR waveform immediately preceding SIB and decrease following SIB
Costescu et al. (2021)	3 participants (3–5 years) / Outpatient clinic	Xiaomi Mi Band 4 Fitness Bracelet	Freq SBP; HR	Wrist	Functional communication training	Nonparametric Wilcoxon test	Elevated HR not associated with emotional outbursts
De Looff et al. (2019)	36 participants (18–57 years) / Outpatient clinic	Empatica E4 wristband	MOAS + ; EDA, BVP, skin temp., movement	Wrist	Natural activities	Multilevel model with epochs nested within the incident level nested within the person level	EDA (PPA and SCL) increase approximately 20 min prior to aggressive behavior
Ferguson et al. (2019)	8 participants (*M* age = 15.9 years) / Outpatient Clinic	Q-Sensor Pod Wristband	Freq SBP; EDA	Wrist or ankle	Natural activities	Visual analysis	Anticipatory increases in EDA 10 min prior to SBP in 60% of cases
Goodwin et al. (2018)	20 participants (*M* age = 10.8 years) / Psychiatric inpatient unit	Empatica E4 biosensor	Freq. SBP; TPA, AOF, ACC, BVP, IBI, EDA	Wrist	Naturalistic observations	Ridge-regularized logistic regression	Predicted aggression in the upcoming 1 min with at least 80% sensitivity
Goodwin et al. (2019)	Same sample as Goodwin et al. (2018)	Empatica E4 biosensor	EDA, HR, motion	Wrist	Naturalistic observations	Ridge-regularized logistic regression	Predict SBPs 1 min in advance
Imbiriba et al. (2020)	Same sample as Goodwin et al. (2018)	Empatica E4 biosensor	EDA, HR, motion	Wrist	Naturalistic observations	Kernel-based nonlinear classification methodology and principal component analysis	Predict SBPs 3 min in advance
Imbiriba et al. (2023)	78 participants (*M* age = 11.9 years) / Psychiatric inpatient unit	Empatica E4 biosensor	EDA, HR, motion	Wrist	Naturalistic observations	Logistic regression; Area under the receiver operating curve	Predicted SBPs 3 min in advance
Lory et al. (2023)	6 participants (3–12 years) / Outpatient clinic	Empatica E4	RRB; HRV	Wrist	Functional analysis of problem behavior	Wilcoxon rank-sum test	Significant positive correlation between RRB duration and HRV
McCabe and Greer (2023)	4 participants (*M* age = 8 years) / Outpatient clinic	Polar H10 HR Monitor	HR	Chest	Functional analysis of problem behavior	Randomization test	No consistent finding between HR and SBP
Nuske et al. (2019)	13 participants (*M* Age = 37.5 months) / Outpatient Clinic	Biopac BioNomadix ECG accelerometer transmitters in pockets	HR, HRV, motion	Back	Contrived tasks (e.g., standing with stranger, waiting for snack)	ANOVA; Conditional logistic regression nested by child; receiver operating curve analysis	HR but not HRV predictive of SBPs
Romanowicz et al. (2023)	10 participants (7–10 years) / Psychiatric inpatient unit	Garmin vivosmart4 smart watch	Coded behavioral state (e.g., Calm, Disruptive); motor activity, HR, and sleep	Wrist	Wore monitor for 24 h / day during admission	Decision tree derived conditional probabilities	Accuracy of predicting SBPs using conditional probabilities was 80.89% (7.2% false positive rate)
Zheng et al. (2021)	7 participants (*M* age = 10.7 years) / Outpatient clinic	Kinect (wall camera), WIINGS motion sensor (sweatshirt), Empatica E4	Head pose, facial expression, motion, BVP, EDA, skin temp	Chest, wrist	IISCA	Random Forest, k Nearest Neighbors, Decision Tree, Neural Network, Support Vector Machine; Discriminant analysis; Naïve Bayes	Random Forest and Neural Network effective; motion involving torso, shoulder, and wrist important

*HR* heart rate, *HRV* heart-rate variability, *EDA* electrodermal activity, *BVP* blood volume pulse, *Freq* frequency, *IISCA* interview-informed synthesized contingency analysis, *SBP* severe behavior problems

## Descriptive Summary of the Studies

### Publication Outlet and Trends

**Fig. 2:**
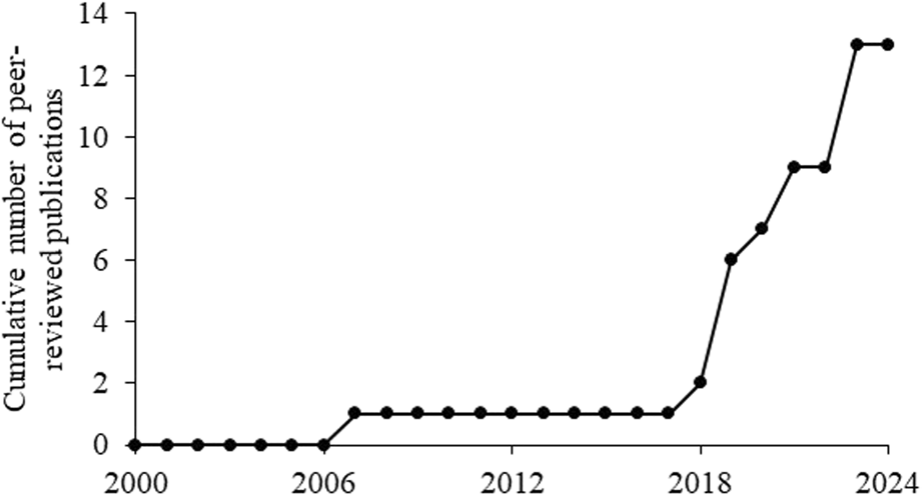
Cumulative Number of Peer-Reviewed Publications Studying Predictive Relations between SBPs and Biometrics

We found each study in this review published their paper in a different academic journal. Five of the articles published their study in a journal catering to professionals working with individuals with NDDs (e.g., *Journal of Applied Behavior Analysis; Advances in Neurodevelopmental Disorders).* The remaining articles were published in journals focused on psychophysiology (e.g., *Journal of Computing Machinery; Sensors).* Figure 2 shows cumulative publications of this line of research since 2000. We chose to present publication occurrence after 2000 to capture publication patterns over the past 25 years. In addition, there have been significant changes in the quality of wearable technology during this period. After 2018, there appears to be a relatively large increase in the number of articles published in this area. As mentioned, this could be related to critical advances in biosensing and machine learning (Rodriguez-Fernandez & Camacho, 2024).

### Participant Demographics and Setting

In total, these projects studied 188 unique individuals (range: 3–78 participants); three studies used the same sample (Goodwin et al., 2018; Goodwin et al., 2019; Imbiriba et al., 2018). The sample size varied considerably by study. The 13 studies included in this review often only reported participant age range. Using this information, these researchers studied individuals from 37.5 months to 57 years with the most common reported age range being between 7 and 16 years. Cognitive abilities varied between Severe Intellectual Disability and borderline cognitive functioning. The setting for five studies was a psychiatric inpatient unit, one study occurred within the participants’ homes, and the remaining seven studies were conducted within an outpatient clinical setting.

### SBPs Evaluated

The SBPs targeted by each study varied from clinically significant problem behavior, such as aggression and self-injury, to subclinical topographies of problem behavior (e.g., negative comments). Although all participants referred to these studies engaged in problem behavior, the actual occurrence of these behaviors during the study varied. For example, although Nuske et al. (2019) recruited 41 participants, only 13 engaged in SBPs for analysis. The participants enrolled in De Looff et al. (2019) likewise displayed a low base rate of SBPs. Although this study enrolled over 100 participants, only 101 aggressive behaviors were included in their analyses. The quality of behavioral observations warrants discussion here, too. Several studies measured reliability by presenting a kappa statistic (e.g., Goodwin et al., 2018) whereas others used interobserver agreement (e.g., McCabe & Greer, 2023). Although use of the kappa statistic is a reasonable approach, interobserver agreement may be preferable in some situations given the low base-rate of SBPs tracked in many of these studies.

#### Wearable Technology

There were a variety of wearable devices used in the 13 studies included in this review. The most common wearable biosensor was the Empatica E4 wrist sensor (7 out of 13 studies). This device afforded researchers data on electrodermal activity (EDA), HR, and motion. Two studies used a shirt that permitted assessment of the heart’s electrical activity via an electrocardiogram and movement from the torso region (Barrera et al., 2007; Zheng et al., 2021) and two studies used a variation of the ActiGraph device (Park et al., 2023). Other devices used included the Garmin Vivosmart4 smart watch, Polar H10 Heart Rate Monitor, Q-Sensor Pod Wristband, and the Xiaomi Mi Band 4 Fitness bracelet. These devices were most often applied to the participant’s wrist (10 of 13 studies). Researchers also placed these wearable devices on participants’ chests, pockets, ankles, or waists. It should be noted that 5 of the 13 studies used a single-sensor approach. That is, these studies only measured one biomarker (e.g., HR) and used that to study SBP occurrence. Given the importance of valid measurements of biometrics within these studies and sensory sensitivities of NDD individuals, it is important to know how well participants tolerated the wearable devices. Many studies did not describe procedures for desensitizing participants to the wearable devices (e.g., Goodwin et al., 2018). The ones that did removed participants that could not tolerate the wearable devices from the study sample (e.g., Imbiriba et al., 2023), reported that they strategically placed the wearable devices in a less accessible place for the participants (e.g., Nuske et al., 2019), or reported no concerns with participant compliance using the wearable device (e.g., McCabe & Greer, 2023).

#### Observation Arrangement

Seven of the studies contrived situations within the clinical setting to observe and study participant SBPs. Four of these studies used an experimental analysis of behavior (e.g., functional analysis of problem behavior), one study evaluated response to an functional communication training evaluation, and two studies contrived play situations. The remaining five studies permitted participants to go through naturally occurring activities on a psychiatric unit or in their homes.

#### Statistical Approaches

The statistical approaches used by these studies varied. Five studies evaluated physiologic change before SBPs without deploying a predictive model (Barrera et al., 2007; De Looff et al., 2019; Ferguson et al., 2019; Lory et al., 2023; McCabe & Greer, 2023). These studies used analytic procedures like visual analysis, percentage difference in HR from before and after SBP occurrence, and nonparametric Wilcoxon test. Of the remaining studies, statistical approaches included logistic regression, Kernel-based nonlinear classification methodology and principal component analysis, and ridge-regularized regression.

#### Outcomes

The 13 studies included in this review each evaluated the extent to which biometrics could predict the occurrence of SBPs. Eleven of the 13 studies identified significant relations between biometrics and the future occurrence of problem behavior. Two of these studies only evaluated HR and showed no relation between changes in HR and the future occurrence of problem behavior (Costescu et al., 2021; McCabe & Greer, 2023). Several studies showed predictors of SBPs occurred 1–3 min prior to the SBP occurrence.

## Critical Evaluation of Predictive Modeling Studies

This review identified 13 studies that claimed to evaluate biometrics predicting the future occurrence of SBPs among NDD individuals. Although 85% (*n* = 11) of these studies reported on biometrics that predicted the future occurrence of SBPs, a little over half actually used methods to validly evaluate this relationship. In particular, of the 11 studies reporting a relationship between biometric measures and SBPs, 6 of these only reported statistical relationships but did not develop predictive models (Barrera et al., 2007; Costescu et al., 2021; De Looff et al., 2019; Ferguson et al., 2019; Lory et al., 2023; Nuske et al., 2019; Romanowicz et al., 2023). Only five studies (Goodwin et al., 2018, 2019; Imbiriba et al., 2020, 2023; Zheng et al., 2021) developed predictive models, of which three (Goodwin et al., 2018, 2019; Imbiriba et al., 2020) used the same data sources. We discuss these studies and examine them critically to ascertain if SBPs can indeed be reliably prospectively predicted. For this, we focus on predictive models that aim to generalize to new individuals (sometimes called population models—e.g., Goodwin et al., 2018), compared to models optimized to individuals. This is because the former are a more stringent test of generalizability to new people.

In Goodwin et al. (2018), youth admitted to a psychiatric inpatient unit connected to wearable motion sensor and engaged in routine milieu activities. These activities included participating in small group therapy, academic lessons, meals in the cafeteria, and leisure time. A direct-care staff collected real-time behavioral data while supporting patient engagement with milieu activities. This arrangement afforded the researchers an opportunity to relate changes in biometric data depending on the (non-) occurrence of problem behavior.

The study used time-series feature extraction from six physiological and physical activity signals (blood volume pulse [BVP], interbeat interval [IBI], electrodermal activity [EDA], acceleration [ACCx, ACCy, ACCz]) collected during naturalistic observations to predict the onset of aggressive behavior. Features were sorted into 15-s bins, including descriptive statistics (e.g., mean, standard deviation, variance) and temporal aggression-related measures (e.g., time since past aggression; TPA). A ridge-regularized logistic regression classifier was then applied to predict whether aggression would occur in a future time window based on features from a preceding time range, using five-fold cross-validation repeated five times for robustness. Prediction performance was evaluated via ROC curves and AUC values across five feature sets: (1) temporal features (TPA); (2) physical activity (ACC); (3) physiological activity (BVP, IBI, EDA); (4) combined physical and physiological features; and (5) all features combined. Results indicated that integrating both physiological and physical activity features yielded the highest predictive performance, suggesting that multimodal time-series data best capture precursors of aggression. Global models (i.e., across all participants) predicting aggression 1 min in advance using all features from the past 180 s achieved an AUC of 0.71. Models incorporating physical and physiological signals consistently outperformed those using temporal features only. Person-dependent models showed higher accuracy, with AUC value of 0.84 indicating improved sensitivity and lower false positive rates compared to global models.

Despite this promising result, a critical decision taken by Goodwin et al. (2018) and Goodwin et al., (2019); which used the same data) was that the cross-validation procedure for the global (i.e., across people models) did not ensure independence in terms of people across training and testing sets. Thus, a different data point, but from the same person, could be in both the training and test sets, meaning that the model could learn person-specific rather than the intended person-general representations.

Imbiriba et al., (2020), which also used the same dataset, reported use of a support vector machine (SVM) classifier which yielded an impressive accuracy of 0.98 for global models (AUC) compared to 0.67 for the logistic regression models used above. In addition to the above issues, the introduction of SVMs includes an additional hyperparameter tuning step, that requires a nested-cross validation approach (i.e., a separate fold for hyperparameter tuning; Booth, 2022) to ensure that there is no “peeking into the data” during the parameter tuning process. Thus, the near perfect result is likely due to even more overfitting to the training data.

Imbiriba et al., (2023) attempted to correct for some of these limitations. These researchers recruited a separate sample of 86 autistic psychiatric inpatients from four psychiatric inpatient units. Similar to Goodwin et al. (2018), these researchers connected the participants to a wearable device to monitor physiological signals during routine unit activities (e.g., meals, therapeutic groups) while their direct-care staff collected data on SBP occurrence. The modeling approach used ridge-regularized logistic regression, support vector machines, and neural networks to predict aggressive behavior in autistic youth based on extracted time-series features from wearable biosensors. At each 15-s interval, models estimated the likelihood of aggression in the upcoming 1–3 min using features from the preceding 1–3 min. Model performance was evaluated primarily using area under the receiver operating characteristic curve (AUROC) across various data-splitting strategies, including a population level leave-individuals-out cross-validation model, which is of interest here.

Of 86 enrolled participants, 70 autistic youth (mean age = 11.9 years; 88.6% male) provided usable physiological and behavioral data across 497 h of naturalistic observation, yielding 6,665 coded episodes of aggression (59.8% self-injury, 31.0% emotion dysregulation, 9.3% aggression toward others). They were able to obtain an impressive AUROC of 0.85 for population models that use person-independent cross-validation where there is strict separation of people in the training and testing sets. However, a closer look indicates a major concern with their computation of the features. In particular, in addition to the physiological measures, they used information from prior aggression episodes in two features—time since past aggression and when a past aggression behavior occurred. Because computation of these features relies on human observations of aggression, this renders the approach semi-automated. More important, given that past behavior is often the best predictor of future behavior, it stands to reason that these features were likely predictive of future aggression episodes. Indeed, across eight experiments, models using augmented feature vectors—which included contextual aggression information—consistently outperformed those using physiological and temporal data alone. The magnitude of the differences was stark. For the population models of interest, the highest accuracy of 0.82 obtained with the augmented features dramatically dropped to 0.56, which barely outperforms chance (0.5).

Zheng et al. (2021) created PreMAC to predict the imminent occurrence of SBPs. PreMAC is a system using data from a Microsoft Kinect to monitor facial expression and head orientation, an Empatica E4 sensor to measure HR, skin conductance, and acceleration, and the Wearable Intelligent Non-invasive Gesture Sensor (WINGS) to measure body movement in an outpatient clinic. While simultaneously connected to the biosensors, participants in Zheng et al. experienced the interview-informed synthesized contingency analysis (IISCA; Hanley et al., 2014). During this experimental functional behavior assessment, a therapist first introduced an antecedent historically associated with the occurrence of targeted problem behavior (e.g., removal of a leisure item to engage in a nonpreferred task). Following the occurrence of problem behavior, the therapist delivered the putative reinforcer (e.g., escape from the nonpreferred task to engage with a leisure item) for a brief period before reintroducing the antecedent. In clinical terms, this experimental FBA leads to identification of response–reinforcer relations that guide development of behavioral treatment (Jessel et al., 2018). In addition to the therapist conducting the IISCA, Zheng et al. arranged for a second therapist to observe the IISCA sessions and collect real-time behavioral data. The combination of the biometric data collected throughout the IISCA and the behavioral data afforded the research team an opportunity to conduct time series analysis to determine significant relations between biometrics and the occurrence of problem behavior.

The PreMAC system used multimodal data from WINGS (body motion), Empatica E4 (physiology and wrist acceleration), Kinect (facial expressions and head movements), and direct behavioral observations to predict precursors to SBPs in autistic children. After filtering, 32 features reflecting motion, physiology, and facial activity were extracted and labeled based on a 90-s window preceding observed precursors. Multiple machine learning algorithms were tested, with Random Forest achieving the highest accuracy (98.51% for individualized models, 82.36% for group models). The team achieved an accuracy of 82% using leave-one-person-out cross-validation. Despite this promising result, there are concerns about the unit of analysis, which consists of an individual multimodal sample rather than an episode of SBP. Further, the modeling approach used a feature selection step (i.e., it is unclear from the description), which would have biased the results because nested-cross-validation (i.e., a separate fold for feature selection; Bosch & D’Mello, 2021) was not performed.

## Discussion

There is a growing body of research studying predictive relationships between biometrics, like HR, and SBP occurrence. This line of research holds significant promise towards creating a safer and more therapeutic environment for a vulnerable client population and their caregivers. This systematic review aimed to examine this nascent literature and to critically examine the extent to which it has delivered on its promise of prospectively predicting SBPs among NDD individuals. Despite the potential of this line of research, there appear to be methodological characteristics that warrant more discussion and lead to interesting avenues for future research.

This literature review identified data from 188 participants. Of these participants, many received diagnoses of ASD, attention-deficit/hyperactivity disorder, or intellectual disability. These participants ranged in age from 3 to 57 years old, although the majority appeared to be between the ages of 7 and 16 years old. It does not seem like age differentially affected study outcome based on a comparison of studies with younger (Costescu et al., 2021) or older (De Looff et al., 2019) participants. We found that all but one study (Park et al., 2023) conducted experimental procedures in a clinical setting. Conducting studies of biosensor readings in controlled clinical settings may be preferred over less-controlled home or community environments (Roos & Slavich, 2023). A clinical setting allows direct relations between biosensor data and human behavior that may be subject to interfering conditions in the natural environment. Given the relative homogeneity of participant ages, diagnoses, and study location, it seems that two natural extensions of this work would be to recruit a more heterogeneous participant population evaluated in the clinical setting and replicating these studies with a similar participant sample in the natural environment (Booth, 2022; Wampfler et al., 2022). This staged approach would allow researchers to change one variable at a time and continue evaluating the durability of the findings thus far.

An estimated one in four adults in the United States use a wearable device on their body (Farivar et al., 2020), with the most common wearable device being wrist-worn devices (e.g., FitBit; Guk et al., 2019; Huhn et al., 2022). In fact, Huhn et al. showed that 138 out of 179 studies included in their systematic review of the literature applied biosensors to participant wrists. It seems to be no surprise that most studies included in this literature review used a wrist-worn device to track key biometrics. Biosensors applied to the wrist have several unique advantages compared to biosensors worn on other parts of the body. For example, these devices may be commonplace in the natural environment (e.g., watches) and more comfortable for participants to wear (Wang et al., 2017). Depending on the biometric being measured, though, applying biosensors to other body areas may be necessary. For example, differences in heart-rate measurement may necessitate placement on the chest (Galli et al., 2022). For now, it seems that most studies attempting to forecast the occurrence of problematic behavior in individuals with NDDs successfully use wrist-worn sensors. The most common product employed by these studies was the Empatica E4 biosensor. This device has research evidence supporting the reliability of data collected from it (Campanella et al., 2023; Schuurmans et al., 2020). If using devices with limited evidence supporting their use, there are published validity assessments to determine if the biometric data being collected is accurate and producing valid data (Van Lier et al., 2020). Future researchers might work to validate other, perhaps more cost effective, biosensors that can support these types of studies.

Beyond the choice of sensor, the experimental arrangement in which behavioral and biometric data are collected may be important. Thus far, studies appear to be mixed in whether they contrive situations for SBP to occur or observe and track naturalistic occurrences of problem behavior. We can see pros and cons to both approaches. Using experimental analysis, like the functional analysis of problem behavior, increases the likelihood that multiple instances of problem behavior could be observed in a short period. In contrast, naturalistic occurrences of problem behavior may afford researchers the opportunity to see the gradual impact of motivating operations on SBP occurrence. It is important to note that naturalistic observations have mostly been studied to determine predictive validity of biometrics and SBPs. The only exception is Zheng et al. (2021), which used the IISCA to develop predictive models of SBP occurrence. Future research may need to study this in more depth to ensure the correct experimental preparations are put into place.

On a similar note, we believe that a key feature to the success of this area of inquiry is designing the appropriate data collection and analytic strategy prior to beginning the study (Neely et al., 2023). There appear to be commonalities in the biometrics measured and the equipment used to measure those biometrics, suggesting emerging methodological standards. In particular, most studies included in this review used advanced statistical techniques to understand the enormous amount of data and its unique complexities. Neely et al. provided an informative discussion about the benefits of “big data” to support the field of behavior analysis. As it relates to the current discussion, behavior analysts may need to collaborate with biostatisticians with specific expertise in machine learning and artificial intelligence approaches to address questions related to prediction of SBPs. Data from accelerometers and heart-rate monitors produce nonlinear information. Although tracking and analyzing behaviors via single-case designs may yield nonlinear relations between independent and dependent variables, the key difference is the size of the data set. Some biosensors will provide up to 32 readings per second. Thus, within a 5-min session, there could be 9,600 (300 s × 32 readings/second) readings of the relevant signal. The specific analytic strategies varied by study. For example, Barrera et al. (2007) use a proportional measure of changes in mean HR before or after problem behavior occurrence whereas Goodwin et al. (2018) utilized a ridge regularized logistic regression model to study their data.

There are benefits to more complex machine learning approaches (e.g., Zheng et al., 2021). These approaches specifically address the issue of prediction and the extent to which biometrics predict the occurrence of SBP. In contrast, approaches such as the randomization test (McCabe & Greer, 2023), visual analysis (Ferguson et al., 2019), and nonparametric Wilcoxon test (Costescu et al., 2021) provide statistical inferences regarding changes in physiologic responding and the occurrence of an SBP. This is an important distinction. The ridge-regularized logistic regression approach potentially handles the issue of “noise” or random changes in behavior that may falsely appear to be related to the outcome variable. Controlling noise and random changes is important to increase the generalizability and validity of findings. Despite the clear benefits to using machine learning approaches, the specific techniques used by authors of these studies (e.g., random forests, gradient boosting) may be less competitive with newer approaches such as multimodal transformers (Xu et al., 2023). For example, the ridge regularized logistic regression approach assumes that the outcome is a linear combination of input features, like HR. This may not be the case for the complex, nonlinear relationships between SBPs and biomarkers. Although certainly not obsolete or outdated, per se, future researchers should consider replicating findings from these studies with newer machine learning techniques.

Irrespective of the choice of approach, across the reviewed literature, methodological considerations limited the external validity, reproducibility, and practical implementation of physiological prediction models for challenging behaviors. Many studies relied on very small samples (e.g., fewer than 10 participants) or narrow subpopulations such as inpatients with severe ASD, preschool children in single classrooms, or psychiatric patients with NDDs. Even in larger enrolled samples, the number of captured high-risk behavioral events is often small, with some analyses based on fewer than 20 episodes, reducing statistical power and precision. Event labeling methods—including live human coding or retrospective staff time stamps—introduce variable delays between actual behavior onset and recorded markers, potentially distorting preincident physiological profiles. Single-sensor approaches dominate, typically focused on wrist-based photoplethysmography (PPG) for HR or EDA, both of which are highly susceptible to motion artifacts and have variable reliability under naturalistic conditions. In predictive modeling, several studies use within-participant training or overlapping time windows without strict temporal blocking, artificially inflating accuracy metrics (e.g., AUROC values > 0.8) and undermining claims of generalizability. Furthermore, functional topographies are often narrow—focusing exclusively on automatically reinforced self-injurious behavior or extreme aggression—leaving uncertain whether models will extend to socially maintained or subthreshold behaviors.

We conclude with some recommendations toward advancing clinically actionable systems. First, the field requires coordinated, large-scale, multisite studies designed to capture a high volume and diversity of target behaviors across settings such as homes, schools, and community programs, in addition to inpatient facilities. These efforts should leverage multimodal sensing arrays combining PPG-derived HR and HRV, EDA, accelerometry, respiration, skin temperature, and potentially environmental/contextual signals (e.g., audio, location tracking) to improve robustness against artifacts and enhance predictive validity. Sensor deployments must be evaluated for comfort, long-term wearability, and minimal reactivity, with explicit quantification of data loss and artifact rates. Predictive models should employ rigorous nested-cross-validation protocols—blocking by both participant and time—to prevent leakage and ensure that performance reflects genuine prospective utility. Finally, prospective field trials are essential to assess operational parameters, such as acceptable false-alarm rates, alert lead times, and the impact of multimodal prediction systems on caregiver decision-making and behavioral outcomes. Without such methodological refinements, the translation of biosensor-based prediction into scalable, real-world prevention tools will remain constrained.

### Short-Term Research

Research questions that could be investigated immediately include studying the effect of experimental arrangement on predictive validity of biometrics on SBPs. Thus far, contriving situations for SBPs to occur is an understudied area of the research. It is a commonplace clinical practice, however. When treating SBPs, behavior analysts often develop rigorous clinical protocols and measure adherence to those protocols (Bergmann et al., 2023). An example protocol to address escape-reinforced SBPs might involve repeated exposure to task demands followed by reinforcement for a brief period (Harding et al., 2009). Because this is how many practitioners teach and reinforce alternative behavior during treatment for SBPs, it will be important to understand whether biometrics predict the occurrence of SBPs in these arrangements better than data collected in naturalistic environments.

Even in inpatient settings, clients may only engage in negative vocalizations or tantrums that include crying and yelling and not SBPs (Romani et al., 2021). Most of the studies described in this systematic review specifically targeted SBPs and the biometrics that might predict its occurrence. Zheng et al. (2021) is an exception because they targeted precursor behavior or less severe behavior that may occur prior to SBPs. Indeed, a growing body of evidence suggests that precursor behavior often occurs prior to SBPs (Fritz et al., 2013; Najdowski et al., 2013). Given that children may not always exhibit SBPs in a clinic setting and it may be clinically indicated to address “precursor” behavior, it is important for studies to see if these predictive patterns are isolated to SBPs or can be captured with other forms of problem behavior.

Finally, it will be important to continue collecting new data sets with more participants engaging in different topographies of problem behavior or coming from diverse backgrounds. This practice may help identify the conditions under which biometrics accurately predict SBPs. As has been done in some cases (e.g., Goodwin et al., 2018), making these data openly available will also support collaboration and transparency in this important line of research (Tincani et al., 2024). Social validity data should also be collected as researchers continue to collect data with participants using wearable technology. As Dovgan et al. (2020) noted, there are a lack of social validity data collected from participants enrolled in behavior-analytic studies using wearable technology.

### Long-Term Research

The potential of this line of research is tremendous. As has been discussed, this research should move toward application. Given that many adults and children alike use wearable devices every day, the possibility of using this technology in practice seems like a real possibility. It remains to be seen whether predictions about the future occurrence of SBPs can potentially be made at least 10–30 s in advance. This time is crucial as it allows a caregiver to engage in antecedent strategies to reduce the likelihood the SBP will occur. For example, if a child variably engages in aggression when directed to complete schoolwork, a 10–30 s warning could allow the caregiver to prompt the child to communicate to escape from that task to prevent the occurrence of SBPs. Reducing the occurrences of SBPs and providing caregivers with a tool to prompt use of preventative strategies could create safer and more therapeutic conditions. On the other hand, it will be important to describe specific methods for how caregivers should respond after receiving an alert indicating the potential occurrence of SBPs. That is, the signal alerting a caregiver to the imminent occurrence of SBPs could inadvertently lead to more punitive intervention, such as restraint or coercion. The ethical implications of these alerts will be important to evaluate.

## Data Availability

No datasets were generated or analysed during the current study.
